# Transgender women, hormonal therapy and HIV treatment: a comprehensive review of the literature and recommendations for best practices

**DOI:** 10.7448/IAS.19.3.20810

**Published:** 2016-07-17

**Authors:** Asa Radix, Jae Sevelius, Madeline B Deutsch

**Affiliations:** 1 Callen-Lorde Community Health Center New York, NY, USA; 2 Department of Medicine, University of California San Francisco, CA, USA; 3 Department of Family and Community Medicine, University of California San Francisco, CA, USA

**Keywords:** hormones, transgender, HIV, antiretroviral therapy

## Abstract

**Introduction:**

Studies have shown that transgender women (TGW) are disproportionately affected by HIV, with an estimated HIV prevalence of 19.1% among TGW worldwide. After receiving a diagnosis, HIV-positive TGW have challenges accessing effective HIV treatment, as demonstrated by lower rates of virologic suppression and higher HIV-related mortality. These adverse HIV outcomes have been attributed to the multiple sociocultural and structural barriers that negatively affect their engagement within the HIV care continuum. Guidelines for feminizing hormonal therapy among TGW recommend combinations of oestrogens and androgen blockers. Pharmacokinetic studies have shown that certain antiretroviral therapy (ART) agents, such as protease inhibitors (PIs), non-nucleoside reverse transcriptase inhibitors (NNRTIs) and cobicistat, interact with ethinyl estradiol, the key oestrogen component of oral contraceptives (OCPs). The goal of this article is to provide an overview of hormonal regimens used by TGW, to summarize the known drug-drug interactions (DDIs) between feminizing hormonal regimens and ART, and to provide clinical care recommendations.

**Methods:**

The authors identified English language articles examining DDIs between oestrogen therapy, androgen blockers and ART published between 1995 and 2015 using PubMed, Cumulative Index to Nursing and Allied Health Literature and EBSCOhost.

**Results and Discussion:**

Published articles predominantly addressed interactions between ethinyl estradiol and NNRTIs and PIs. No studies examined interactions between ART and the types and doses of oestrogens found in feminizing regimens. DDIs that may have the potential to result in loss of virologic suppression included ethinyl estradiol and amprenavir, unboosted fosamprenavir and stavudine. No clinically significant DDIs were noted with other anti-retroviral agents or androgen blockers

**Conclusions:**

There are insufficient data to address DDIs between ART and feminizing hormone regimens used by TGW. There is an urgent need for further research in this area, specifically pharmacokinetic studies to study the direction and degree of interactions between oral, injectable and transdermal estradiol and ART. Clinicians need to be vigilant about possible interactions and monitor hormone levels if concerns arise. More research is also needed on the provision of hormone therapy and gender-affirming care on the long-term health outcomes of HIV-positive TGW.

## Introduction

### HIV prevalence among transgender women

Transgender individuals are those whose gender identities and/or gender expression do not fully align with their assigned sex at birth. Many cultures around the world recognize transgender people using varied terms such as “Kathoeys” in Thailand [1]; “Hijras” in India, Bangladesh and Pakistan [2–4]; “Warias” in Indonesia [5]; the “Rae Rae's” and “Mahu's” in French Polynesia [6]; and “Travestis” of Latin America [7]. Currently, there are no national-level estimates of the prevalence of transgender identity for any country, although India, Bangladesh, Nepal and Pakistan have recently added a third gender option to the census. Approximately 0.5% of adults (aged 18–64) who participated in the Massachusetts Behavioral Risk Factor Surveillance System self-identified as transgender when a gender identity question was included in the survey [8]. The lack of inclusion of gender identity in national census data in most countries means that the true size of the transgender population is unknown, confounding public health efforts to fully understand the extent of HIV burden within these populations.

Despite the fact that few countries use HIV surveillance methods that permit the identification of transgender people, global estimates have demonstrated an increased HIV burden among transgender women (TGW), with a pooled HIV prevalence of 19.1%, and an odds of HIV among transwomen 48 times that of the general population [9]. Although limited data exist on HIV incidence, two studies demonstrate incidence among TGW of 3.4 to 7.8 per 100 person-years, placing them as the group at highest risk for HIV infection [10,11].

### TGW and the HIV continuum of care

TGW face a complex array of psychosocial challenges that complicate their access and adherence to HIV care, such as limited access to and avoidance of health care because of stigma and past negative experiences with providers, prioritization of gender-related health care and concerns about adverse interactions between antiretroviral therapy (ART) medications and hormone therapy [12,13]. Social and economic marginalization because of transphobia (negative societal attitudes towards transgender persons) often result in poverty and unstable housing, familial alienation, limited formal education, limited social support, mental illness, trauma and victimization, substance abuse and introduction to sex work often at an early age [14–20]. These factors can result in late or no presentation to HIV medical care and poor health outcomes [21].

Data on the HIV care continuum among TGW are limited because both informational and institutional erasures render many TGW invisible in data collection and research [22]. HIV testing rates reveal that many HIV-positive TGW are less likely than the general population to be aware of their HIV status despite their elevated HIV risk [23]. Melendez *et al*. also showed disparities with ART at one clinical site, with fewer TGW (59%) than non-transgender people (82%) taking ART [24]. Even when receiving ART, TGW have been shown to be significantly less likely to report optimal adherence [25]. A study examining community viral load in San Francisco demonstrated that TGW had a higher community viral load, almost three times compared to non-transgender persons, consistent with low rates of ART [26]. A study evaluating the care continuum among newly diagnosed TGW in San Francisco revealed that 77% reported being linked to primary care within three months of their HIV diagnosis, 65% were taking ART, but only 44% were virologically suppressed (viral load ≤200 copies/mL) [27]. This last study likely represents a “best-case scenario” because many TGW may not be engaged in care [13], but the indicators still fall short of the UNAIDS 90-90-90 targets [28].

### Hormone therapy for TGW

Hormone therapy is one aspect of gender-affirming care that is utilized as part of medical transition. Gender affirmation in clinical settings goes far beyond hormones and includes the creation of a welcoming environment, including the use of patients’ preferred names and pronouns and providers who are knowledgeable about transgender health issues [29]. The desire for gender-affirming health care, such as hormone therapy, is a critical factor that may both serve as an adjunct to and require special considerations for effective engagement in HIV medical care. Anecdotal reports from health care providers indicate that hormone treatment can be an incentive for TGW to seek and adhere to ART [30].

The goal of feminizing hormone therapy is to induce the secondary sex characteristics of the affirmed gender and to reduce the sex characteristics of the individual's natal sex. For TGW, feminizing regimens usually involve the use of an androgen blocker in addition to oestrogen, with many regimen options available, as outlined in Table 1. Feminizing hormonal regimens result in favourable effects, including breast growth, softening of the skin, slowing of androgenetic hair loss and fat redistribution, however will not have an effect on beard hair or voice, for which electrolysis/laser treatment and voice therapy are recommended [31,32].

**Table 1:** Examples of oestrogens, androgen blockers, routes and dosing used in feminizing hormone regimens

	Route	Dose
**Hormone**		
Estradiol/estradiol valerate	Oral or sublingual	2–8 mg daily
Estradiol valerate	Intramuscular	20–40 mg every 2 weeks
Estradiol cypionate	Intramuscular	2 mg every week or 5 mg every 2 weeks
Estradiol gel Topical	Topical	0.75 mg two to three times daily
Estradiol patch transdermal	Transdermal	25–400 µg
Conjugated equine oestrogens (not recommended – see text)	Oral	
Ethinyl estradiol (not Recommended – see text)	Oral	
**Anti-androgen**		
Spironolactone	Oral	50–400 mg daily
Finasteride	Oral	2.5–5 mg daily
Cyproterone acetate	Oral	50–150 mg daily
Goreselin	Subcutaneous	3.6 mg/month or 11.25 mg/3 months
Leuprolide acetate	Intramuscular	3.75 mg/month

Sources: Adapted from Royal College of Psychiatrists [33], Hombre *et al*. [31] and the Blueprint for the provision of comprehensive care for trans people and trans communities in Asia and the Pacific [34].

In the United States, spironolactone, a mineralocorticoid-receptor antagonist with anti-androgen properties, is most frequently used for androgen blockade [31]. Cyproterone acetate and gonadotropin-releasing hormone (GnRH) agonists are the androgen blockers most frequently used in Europe [33,35]. Oestrogen may be administered by oral, intramuscular or transdermal routes [31]. The World Professional Association of Transgender Health recently published the latest recommendations for the health of transsexual, transgender and gender non-conforming people, and these guidelines are widely used in the USA and internationally [32]. Several countries have also published their own guidelines, to reflect the local availability and acceptability of hormonal formulations as summarized in Table 2.

**Table 2:** Selected international guidelines for transgender health care

Agency	Year	Guideline
The Endocrine Society, USA [31]	2009	Endocrine treatment of transsexual persons: an Endocrine Society clinical practice guideline
The World Professional Association of Transgender Health (WPATH) [32]	2011	Standards of care for the health of transsexual, transgender, and gender-nonconforming people, Version 7
Counties Manukau District Health Board, Wellington, New Zealand [36]	2011	Gender reassignment health services for trans people within New Zealand. good practice guide for health professionals
Royal College of Psychiatrists, London, UK [33]	2013	Good practice guidelines for the assessment and treatment of adults with gender dysphoria, 2013
Pan American Health Organization [37]	2014	Blueprint for the provision of comprehensive care for trans persons and their communities in the Caribbean and other Anglophone countries
Health Policy Project, Asia Pacific Transgender Network, United Nations Development Programme [34]	2015	Blueprint for the provision of comprehensive care for trans people and trans communities in Asia and the Pacific
Center of Excellence for Transgender Health, University of California, San Francisco	2016	Guidelines for the primary care of transgender, gender nonconforming, and gender non-binary people

### Utilization of feminizing hormonal therapy among TGW

The proportion of TGW who opt to start hormones varies significantly across countries because of the availability of oestrogens and androgen blockers, cost and whether these are available only through medical sources or without prescriptions. In addition, some TGW opt not to use hormones for personal/identity reasons or medical contraindications. The highest rates of hormone use are in countries where hormones are readily available without prescription, and where a greater level of acceptance exists towards transgender persons, for example, Thailand, where studies report hormone use among TGW of 73 to 94%, mostly obtained without a prescription [38–40]. In the USA and Canada, hormone utilization among TGW is reported to be 27 to 93% [23,41–45], with up to 60% obtaining their hormones outside of the medical system [44,46–48]. The lowest rates of non-prescription hormone use are reported in Canada and the UK, two countries where hormones are available through the health care system at no expense to the transgender client [46,49]. Use of non-prescription hormones is associated with less knowledge about clinical guidelines and adverse effects of hormones [49,50]. Users of non-prescription hormones usually do so because of difficulty accessing gender-affirming care and are therefore less likely to be monitored appropriately and to use optimal regimens [44,50]. Both ethinyl estradiol (a potent oestrogen and a component of oral contraceptives, or OCPs) and conjugated equine oestrogens are no longer recommended as feminizing agents because of the increased risk of venous thromboembolism [51,52]. Outside of health care settings these agents are often used by TGW for medical transition [37,50].

### Comorbidities and other considerations

Since the advent of ART, people living with HIV are living longer, healthier lives. There has been an increase in awareness of some of the age-related health conditions that are more prevalent in this population that hold particular considerations for HIV-positive TGW receiving hormone therapy.

Low bone mineral density (BMD) has been reported to occur more frequently in HIV-positive individuals [53,54] as well as HIV-negative individuals receiving tenofovir disoproxil fumarate (TDF) for pre-exposure prophylaxis (PrEP) [55]. This is a particular concern for TGW because hormones have a direct effect on bone metabolism. There is evidence that the risk of osteopenia and osteoporosis may be elevated in TGW even before initiation of hormone therapy [56]. Reasons include reduced engagement in sports resulting in lower muscle mass and grip strength and lower levels of vitamin D levels [56]. After starting feminizing regimens there are inconsistent data about risk for osteopenia, with studies showing increase, decrease or no change in BMD [57–59]. The differences in results may be because of the regimens used (some used unopposed androgen blockers for a period of time before initiating hormones) and length of follow-up. Known risk factors for osteoporosis include underutilization of hormones after gonadectomy or use of androgen blockers without or with insufficient oestrogen [31]. GnRH agonists also may result in short-term decline in BMD [60].

Cardiovascular disease (CVD) is another area of special concern for TGW living with HIV. A recent prospective study demonstrated increased CVD mortality in this population [61]. Feminizing hormone therapy may be an independent risk factor for CVD; however, this population also experiences higher rates of other CVD risk factors including tobacco use, obesity, diabetes and dyslipidemias [61–67]. The already well-documented high rates of CVD among people living with HIV make this an additional concern for HIV-positive TGW receiving hormones. In the absence of clinical trials evaluating health outcomes associated with different hormone regimens, clinicians have advocated the use of transdermal oestrogens in those with a history of CVD or a predominance of risk factors, based on studies conducted in postmenopausal women [68,69].

### Guidelines for ART

The goals of HIV treatment are to improve health outcomes for people living with HIV and to reduce HIV transmission. There are five classes of antiretroviral agents that have different mechanisms of action during the HIV life cycle. Effective treatment regimens usually consist of ART with at least three agents. The initial regimen usually includes three agents from two classes, most often two nucleoside reverse transcriptase inhibitors (NRTIs) with either a non-nucleoside reverse transcriptase inhibitor (NNRTI), a protease inhibitor (PI), which is usually “boosted” with ritonavir, or an integrase strand transfer inhibitor (INSTI). Two other antiretroviral agents block entry of HIV into human cells using different mechanisms, the CCR5 receptor antagonists and fusion inhibitors.

The recent update in WHO HIV treatment guidelines, expanding ART to all individuals regardless of CD4 cell count, means that an additional 9 million individuals, including many transgender individuals, are now eligible for ART. It is extremely timely therefore to determine what impact, if any, hormone therapy has on the uptake as well as adverse effect profile of ART.

The recommendations for initial antiretroviral regimens vary globally based on considerations such as cost, availability of generic agents, adverse effect profile and prevalence of comorbidities. The WHO guidelines recommend an initial regimen of two NRTIs, either lamivudine (3TC) or emtricitabine (FTC) with TDF and the NNRTI efavirenz (EFV), or if not available, nevirapine (NVP) [70]. Other NRTI options to use in place of tenofovir include zidovudine (ZDV) [70]. In the USA, the two guidelines predominantly used are the International Antiviral Society-USA Panel and Department of Health and Human Services (DHHS) Panel on Antiretroviral Guidelines for Adults and Adolescents [71,72]. Both guidelines recommend the use of INSTIs, such as dolutegravir or elvitegravir in initial regimens, as well as the PI darunavir, in combination with two NRTIs (TDF, FTC or 3TC); however, minor differences exist in the recommendations for other agents.

### TGW and PrEP

The use of emtricitabine and TDF for PrEP is an important biomedical intervention in preventing HIV acquisition. A recent substudy of the iPrEx trial revealed that TGW were less likely to have detectable drug levels compared to non-transgender MSM [30]. Furthermore, TGW may have concerns about potential interactions between feminizing hormone regimens and PrEP that reduce the likelihood of PrEP uptake among HIV-negative TGW [25].

### Potential interactions between antiretroviral regimens and feminizing hormone regimens

The current guidelines for transgender care listed in Table 2 do not address interactions between ART and hormonal therapy; however, potential interactions may exist.

Many antiretroviral agents are metabolized through the cytochrome P450 (CYP) system [72], allowing for potential drug-drug interactions (DDIs) with other medications that use the same pathway and often resulting in unpredictable changes. Most of the studies that examine for interactions between antiretrovirals and oestrogens have focused primarily on OCPs, because the predominant concern has been effects on the efficacy of contraception in HIV-positive non-TGW. Ethinyl estradiol, the main component of OCPs, is predominantly metabolized through the cytochrome P450 3A4 (CYP3A4) enzyme pathway, presenting concerns for interactions with the NNRTIs efavirenz and nevirapine, which are CYP3A4 inducers, and the PIs, which are metabolized by and known to be potent CYP3A4 inhibitors [73]. Understanding the potential for interactions is important because untoward increases in oestrogen or antiretroviral drug levels may cause serious adverse effects, whereas those that reduce these drug levels may result in loss of virologic suppression or inadequate feminization. In qualitative studies, TGW have reported fears that ART can limit the effect of hormones, a serious concern for this population [12]. Faced with this dilemma, TGW have reported that they may prioritize gender-affirming health care over HIV treatment [12] which underscores the importance of having evidence-based data on DDIs.

## Methods

To identify studies investigating antiretroviral agents and the components of feminizing hormone regimens, the authors retrieved English language articles from 1995 to 2015 using PubMed (MEDLINE), Cumulative Index to Nursing and Allied Health Literature (CINAHL) and EBSCOhost. Medical subject headings (MeSH) terms for ART (“Antiretroviral,” “HAART,” “Anti-HIV Agents”) were cross-referenced with feminizing hormonal agents (including “estrogen,” “estradiol,” “hormonal contraceptives”) and androgen blockers (“spironolactone,” “finasteride,” “gonadotropin releasing hormone agonist,” “GnRH,” “leuprolide,” “goreselin”). Androgen blockers were also cross-referenced with “interactions.” The authors reviewed abstracts for relevance, with subsequent full-text review for data abstraction. Inclusion criteria were primary DDI and pharmacokinetic studies that evaluated interactions between antiretroviral agents and oestrogens or androgen blockers. Additional information was obtained from pharmacokinetic data listed in package inserts for antiretroviral agents and named anti-androgens, the University of Liverpool's HIV drug interactions website [74] and the DHHS guidelines for the use of antiretroviral agents in HIV-1-infected adults and adolescents [72].

## Results and discussion

### Feminizing hormones

The authors reviewed 165 unduplicated articles that included references to feminizing hormonal agents. These were reviewed for relevance, resulting in identification of 26 peer-reviewed articles, of which 8 were reviews [73,75–81]. Of the remaining 17 articles, one involved an ART agent no longer in use [82] and one was for an investigational drug [83]. The remaining 16 articles were used in this review [84–99]. All but one (an *in vitro* study) [99] were *in vivo* pharmacokinetic DDI studies. All of the available studies evaluated ART interactions with OCPs and not with other feminizing hormones. The studies were all conducted in non-TGW, with 10 to 34 participants. In all but five studies [87,88,95,96,98], the participants were all HIV-negative.

There are few studies that have examined interactions between exogenous oestrogens and ART, and these have all investigated effects of OCPs [76]. Although we can speculate about the direction of interactions based on these data, they may not reflect the true interactions seen using the types and doses of oestrogens used in feminizing regimens. One review found that many studies of PIs and NNRTIs showed inconsistencies in the direction and level of interactions, mainly because of differences in study design and OCP regimen [76]. Table 3 summarizes all known effects of ART on ethinyl estradiol. The only known interactions of ethinyl estradiol on ART that have the potential to result in loss of virologic suppression are with amprenavir, unboosted fosamprenavir and stavudine [72,99], although the latter was a single *in vitro* study in peripheral blood lymphocytes. It may be prudent however to recommend that these ART drugs be avoided in the treatment of TGW receiving feminizing hormones.

**Table 3:** Interactions between antiretroviral therapy and ethinylestradiol

Effect on ethinyl estradiol levels (AUC)	Antiretroviral	Change
Increase	Atazanavir [72] Etravirine [89] Fosamprenavir [72] Rilpivirine [72,90]	AUC ↑ 48% AUC ↑ 22% Cmin ↑ 32% AUC ↑ 0–14%, Cmax↑ 17%
Decrease	Atazanavir/ritonavir [72,84] Darunavir/ritonavir [86] Fosamprenavir/ritonavir [84] Lopinavir/ritonavir [72,87] Nevirapine [72,88] EVG/c/TDF/FTC [72] Tipranavir/ritonavir [72]	AUC ↓ 19%, Cmax ↓ 16% and Cmin ↓ 37% AUC ↓ 44%, Cmin ↓ 62%, Cmax ↓ 32% AUC ↓ 37%, 28% ↓ Cmax and 34% AUC ↓ 42%, Cmax ↓ 41%↓58% AUC ↓ 29% AUC ↓ 25%, Cmin ↓ 44% AUC ↓ 37 to 48%
No effect	Dolutegravir [72,97] Efavirenz [94] Maraviroc [91] Raltegravir [72,92] Tenofovir [94] Zidovudine [95]	
No data	Abacavir Atazanavir/cobicistat Darunavir/cobicistat	

### Anti-androgens

There were no published pharmacokinetic studies that investigated interactions between ART and spironolactone or finasteride. Drug package inserts, the DHHS guidelines and the HIV drug interactions website also did not flag potential drug interactions with these agents. Finasteride is an inhibitor of type II alpha-reductase, blocking conversion of testosterone to 5-alpha-dihydrotestosterone (DHT). Although finasteride is primarily metabolized through the CYP3A4, it has no effects on the cytochrome P450 system [100,101]; therefore, it is unlikely to have an effect on ART levels. When finasteride is co-administered with etravirine, efavirenz or nevirapine, this may result in decreased finasteride levels, but the clinical significance of this is unknown [74]. Spironolactone, the agent most often used in feminizing regimens in the USA, is also metabolized through the cytochrome P450; however, no relevant DDIs occur with ART through this mechanism [74]. The GnRH agonists leuprolide and goreselin are both metabolized through intravascular and extravascular hydrolysis of the C-terminal amino acids followed by excretion in the urine [102,103]. No pharmacokinetic-based DDI studies have been conducted with these agents [102,103].

A special consideration for HIV-positive TGW is that anti-androgens may be unnecessary for medical transition purposes because HIV infection is frequently associated with low testosterone levels. Studies of HIV-positive natal men have shown a prevalence of hypogonadism of approximately 25% [104]. Before the initiation of hormonal therapy, total testosterone levels should be checked.

This review did not find evidence of clinically significant interactions between ART and feminizing hormones (ethinyl estradiol) for medical transition. Although the possible increase in CVD and osteoporosis among TGW living with HIV is concerning, it underscores the importance of engagement in HIV primary care, which as noted above could be enhanced by provision of hormone therapy and other gender-affirming services. Engagement in primary care services will allow for appropriate health care screenings as well as identification and reduction of modifiable cardiovascular risk factors, such as tobacco use, hyperlipidemia, overweight and obesity.

In fact, bundling HIV care with hormone and other aspects of gender-affirming care may result in synergistic enhancements, as has been demonstrated in other medical contexts. A preliminary data analysis, led by MD, of the ongoing HRSA SPNS Transgender Women of Color initiative found associations between current ARV use, undetectable viral load and receipt of HIV primary care in the past six months and receiving a hormone prescription from the participant's HIV primary care provider (as opposed to other sources).

## Conclusions

Although the data investigating oestrogens and ART come mainly from OCP data, it is likely that, apart from amprenavir, unboosted fosamprenavir and possibly stavudine, no clinically significant interactions exist between feminizing regimens and ART. These data are also important for HIV prevention among at-risk HIV-negative TGW, who may not be inclined to use PrEP until clear information is available about potential interactions between TDF and feminizing hormone regimens [105].

Clinicians should maintain vigilance for perceived or actual interactions between hormone therapy and ART, and take appropriate clinical or patient educational action as indicated. This may include monitoring estradiol levels while on ART in order to assess for elevated or subtherapeutic levels as well as ongoing viral load monitoring.

Clinicians should be aware of the possible increase in CVD and osteoporosis among HIV-positive TGW on hormones and seek to identify and reduce modifiable risk factors.

There is an urgent need for further research, specifically pharmacokinetic and pharmacodynamics studies to evaluate for interactions between oral, injectable and transdermal estradiol and ARTs. More research is also needed on the impact of hormone therapy on long-term health outcomes of HIV-positive TGW, not only to evaluate adverse effects but also to assess the potential positive impact of hormones on HIV-specific outcomes, like retention and engagement in care and virologic suppression as well as other preventive health needs.

The key limitation to this review is the lack of evidence-based data to support recommendations for TGW living with HIV. Meaningful inclusion of sexual orientation and gender identity data in national surveillance systems, as well as prospective studies investigating the long-term health outcomes for transgender people accessing medical transition services, will allow for more robust data upon which to build guidelines for care.
